# Editorial: Brain connectivity in neurological disorders

**DOI:** 10.3389/fnsys.2023.1274801

**Published:** 2023-09-26

**Authors:** Alessandro Salvalaggio, Lorenzo Pini, Alessandra Griffa, Maurizio Corbetta

**Affiliations:** ^1^Padova Neuroscience Center, University of Padua, Padua, Italy; ^2^Department of Neuroscience, University of Padua, Padua, Italy; ^3^Leenaards Memory Center, Department of Clinical Neurosciences, Lausanne University Hospital, Lausanne, Switzerland; ^4^Veneto Institute of Molecular Medicine (VIMM), Padua, Italy

**Keywords:** brain connectivity, disconnection, clinical, diseases, structural connectome, functional connectome

The exploration of brain network connectivity has allowed us to unravel the complex functional and structural architecture of the human brain, uncovering its intricate composition of interconnected modules and networks. These groundbreaking studies have shed light on how various neurological and psychiatric disorders can be considered as “disconnectivity syndromes,” paving the way for the identification of new biomarkers to aid in the diagnosis and treatment of these diseases. However, the clinical application and impact of these findings have fallen short of expectations. Currently, connectivity measures are not integrated into the clinical assessment of neurological and psychiatric patients, nor are they employed as surrogate markers in clinical trials. Nonetheless, with the substantial body of evidence available, it is crucial to seize this opportunity and translate these findings into practical applications in the clinical field. The aim of this Research Topic consists in collecting studies applying connectivity methods in different clinical populations alongside the hypothesis that neurological disorders are (at least partially) mediated by connectivity alterations.

Among the studies herein collected, case reports focusing on uncommon clinical presentation, offer valuable insights into the value of connectivity approach at the individual patient level. In a study by Monai et al., a patient with subclinical cognitive deficits across multiple domains, recurrent delirium, and a focal frontal lesion was examined using a multi-modal approach. By integrating various types of disconnections—including electroencephalography (EEG), functional and structural disconnectivity, and metabolism—the researchers found how brain dysfunction extended beyond the focal lesion, matching with cortical glucose hypometabolism and therefore justifying the broad clinical presentation. In another case report, Indovina et al. described a patient who developed agoraphobia after the surgical removal of a glioma located in the right parietal cortex. The researchers reported extensive post-surgery reorganization within the vestibular network, as evidenced by changes in both structural and functional connectivity measures, thus helping in understanding the pathophysiology underlying the occurrence of agoraphobic symptoms. Overall, these case reports demonstrate the feasibility of applying connectivity analyses to individual subjects in a clinical setting thus providing an additional tool for the diagnosis and treatment. Brain connectivity approach may also provide biomarkers of cognitive impairment in multiple sclerosis, which is the most debilitating neurological disease among young adults. Several studies have already demonstrated a correlation between alterations in brain connectivity and the clinical severity of MS. Building upon these findings, Grothe et al. provided further evidence about the close relationship between processing speed performance and the structural connectivity of frontoparietal regions. Interestingly, connectivity changes may also appear (and be measured) when the damage is outside the central nervous system. Quettier et al. successfully employed an EEG-based approach to identify connectivity modulations in individuals with seventh cranial nerve damage. Specifically, their study revealed a reduced strength of connectivity between sensorimotor and visual regions in participants affected by facial palsy when compared to the matched controls.

Recent advancements have been made in the analysis of connectivity data, offering promising results and expanding our understanding of clinical information derived from connectivity. Spadone et al. conducted a dynamic functional connectivity study on stroke patients, introducing a novel functional dynamic approach. This analysis method examines the signal in terms of transient conditions of neural network reconfigurations. The findings from this study revealed that strokes leading to spatial attention deficits impact the temporal configuration of functional connections. The altered connectivity patterns were found to be associated with the severity of spatial neglect.

However, while these studies are intriguing, their practical application in clinical settings is limited due to various challenges. These include the complexity of performing comprehensive connectivity examinations, the patient's limited compliance for long acquisition time, the requirement for advanced processing and analysis skills, and the lack of access to clinical facilities. These limitations have hindered the widespread translation of these findings into clinical practice. In recent years, researchers have developed alternative approaches to assess brain disconnection. These approaches aim to overcome the need for extensive data acquisition and processing by utilizing a publicly available normative dataset. These methods have primarily been developed within the context of brain focal lesions, where the volume of the lesion is integrated into a normative functional or structural connectome allowing to estimate which regions or tracts have been likely disconnected by the pathology (Boes et al., 2015; Foulon et al., 2018). Implementing these approaches in the clinical assessment of brain lesions holds great potential. Nabizadeh and Aarabi conducted a comprehensive review of the recent literature in this field, highlighting the growing body of research. Their review revealed that more than fifty papers have been published recently, further substantiating the interest and progress in this area of study. Within this evolving context Klingbeil et al. conducted a comprehensive analysis of 270 stroke patients to assess the impact of post-stroke depressive symptoms in relation to structural and functional indirect disconnections. They identified a significant association between higher depression scores and both lesions topology and white-matter structural disconnection in the right temporal lobe. No significant associations were observed with functional disconnections. These findings indicate that in the context of stroke, structural disconnection may exert a more preeminent predictive role compared to functional disconnections, which aligns with recent findings (Salvalaggio et al., 2020). Finally, Sansone et al. investigated the pattern of network involvement of glioblastoma (GBM), reporting a preferential overlap between GBM and specific networks suggesting that tumor growth and spreading might not be independent of brain activity, although, network-topology information is overall scarcely informative about overall survival in these patients.

In conclusion, this Research Topic suggests that connectivity approaches might have the potential to be widely implemented in the clinical framework, despite several limitations which should be addressed by future research.

## Author contributions

AS: Writing—original draft, Writing—review and editing. LP: Writing—original draft, Writing—review and editing. AG: Writing—review and editing. MC: Writing—review and editing.
